# A metric learning-based method for biomedical entity linking

**DOI:** 10.3389/frma.2023.1247094

**Published:** 2023-12-19

**Authors:** Ngoc D. Le, Nhung T. H. Nguyen

**Affiliations:** ^1^Faculty of Information Technology, University of Science, Ho Chi Minh City, Vietnam; ^2^Vietnam National University, Ho Chi Minh City, Vietnam; ^3^Department of Computer Science, School of Engineering, University of Manchester, Manchester, United Kingdom

**Keywords:** metric learning, imbalanced data, biomedical entity linking, entity normalization, triplet loss, soft-radius clustering

## Abstract

Biomedical entity linking task is the task of mapping mention(s) that occur in a particular textual context to a unique concept or *entity* in a knowledge base, e.g., the Unified Medical Language System (UMLS). One of the most challenging aspects of the entity linking task is the ambiguity of mentions, i.e., (1) mentions whose surface forms are very similar, but which map to different entities in different contexts, and (2) entities that can be expressed using diverse types of mentions. Recent studies have used BERT-based encoders to encode mentions and entities into distinguishable representations such that their similarity can be measured using distance metrics. However, most real-world biomedical datasets suffer from severe imbalance, i.e., some classes have many instances while others appear only once or are completely absent from the training data. A common way to address this issue is to down-sample the dataset, i.e., to reduce the number instances of the majority classes to make the dataset more balanced. In the context of entity linking, down-sampling reduces the ability of the model to comprehensively learn the representations of mentions in different contexts, which is very important. To tackle this issue, we propose a metric-based learning method that treats a given entity and its mentions as a whole, regardless of the number of mentions in the training set. Specifically, our method uses a triplet loss-based function in conjunction with a clustering technique to learn the representation of mentions and entities. Through evaluations on two challenging biomedical datasets, i.e., MedMentions and BC5CDR, we show that our proposed method is able to address the issue of imbalanced data and to perform competitively with other state-of-the-art models. Moreover, our method significantly reduces computational cost in both training and inference steps. Our source code is publicly available here.

## 1 Introduction

Entity linking, also known as named entity normalization, aims to disambiguate the meaning of entities occurring in free text. In the biomedical domain, the task involves mapping biomedical entities (e.g., diseases, genes, drugs, and chemicals) to concepts (or *entities*) in a standardized ontology or a biomedical knowledge base [e.g., the Unified Medical Language System (UMLS) Bodenreider, 2004]. This is an essential step for many downstream tasks, including relation extraction (Xu et al., 2016; Li et al., 2017), information retrieval, question answering (Kim et al., 2019), and knowledge base construction (Wawrzik et al., 2023).

The task of biomedical entity linking presents a number of challenges, the first of which concerns the ambiguity and diversity of biomedical entities. For example, although “battens disease” and “juvenile cerebroretinal degenerations” have highly different surface forms, they both refer to the same disease entity that causes vision loss, motor dysfunction and dementia. Meanwhile, “testosterone injection” may refer either to a therapeutic or preventive procedure, or a clinical drug, meaning that, depending on its specific textual context, it should be mapped to one of two different concepts. Such phenomena mean that automatic entity linking models may fail to correctly disambiguate entities if they cannot sufficiently interpret the context in which the entities occur.

Recent studies have addressed the problem using metric learning methods (Liu et al., 2020; Agarwal et al., 2021; Bhowmik et al., 2021). These methods firstly learn representations of mentions and their contexts, as well as representations of entities, in a latent space. They then use a similarity metric to select the entity with the highest similarity score to the mention. The performance of these methods heavily depends on the model architecture and sampling strategy employed. Experimental results show more complex models achieve the best performance, since they can learn better mention and entity representation (Agarwal et al., 2021). Other studies based on generative models also show promising results, e.g., GENRE (Cao et al., 2020) and (Yuan et al., 2022). However, these state-of-the-art (SOTA) models require a large amount of computational resources for training and require significant time to perform inference. In this study, we employ the former approach, using a dual-encoder architecture to learn representations of mentions and entities. This architecture reduces computational cost during the training phase and allows indexing for later retrieval, which reduces inference time compared to cross-encoder architectures.

A further challenge of biomedical entity linking relates to the problem of data imbalance, i.e., some concepts have many mentions in the training data, while other concepts only have a handful of mentions. Although data imbalance is often severe in real-world entity linking datasets, especially in the biomedical domain, no specialized algorithms have been designed to deal with it. A common solution to the data imbalance issue is to down-sample the dominant classes to make the number of samples across different classes more well-balanced (Dubey et al., 2014). However, down-sampling may result in information loss. For example, synonyms or variants of entities, which constitute important evidence for entity linking, may be removed during down-sampling.

To alleviate the data imbalance issue, we propose a new loss function based on triplet loss (Schroff et al., 2015), namely *prototype-based triplet loss*, which concurrently learns the representations of all mentions that refer to the same entity (class) via the centroid of its cluster. We further boost the performance of the model by using soft-radius neighbor clustering inspired by Bentley (1975) to detect latent clusters inside a class, since mentions in a class do not always come from a single distribution.

We evaluate our proposed methods on two biomedical datasets, i.e., MedMentions (Mohan and Li, 2019) and BC5CDR (Li et al., 2016). Our experimental results indicate that by using the prototype-based triplet loss, our system is able to outperform the use of traditional triplet loss, both with and without the application of the down-sampling. Moreover, combining the proposed triplet loss with soft-radius neighbor clustering results in performance that is competitive with other SOTA methods on both experimental datasets.

## 2 Background and related work

### 2.1 Metric learning

Metric learning is a sub-field of supervised machine learning, which aims to learn to distinguish samples based on the distance between them (Davis et al., 2007; Hoffer and Ailon, 2015). Specifically, in a representation space, there should only be a small distance between the vectors of two samples that belong to the same class. Conversely, the distance between the vectors of two samples belonging to different classes should be larger. In recent years, metric learning has been shown to be effective in a number of computer vision tasks, such as image retrieval (Zhong et al., 2021), object recognition (Sohn, 2016), and face recognition (Cao et al., 2013), and also for natural language processing tasks, such as text classification (Wohlwend et al., 2019) and entity linking (Liu et al., 2020).

The performance of metric learning methods is dependent on three factors, i.e., model architecture, objective function, and sample selection. Model architectures can be classified into interaction-based and representation-based approaches. The interaction-based approach (Wan et al., 2015) builds local interactions between two samples and learns hierarchical interaction patterns to match them. Meanwhile, the representation-based approach (Dong and Shen, 2018) consists of two components: (1) an encoder, which transforms samples into embeddings and (2) a distance function, which computes the similarity between pairs of embeddings from the encoder.

Normally, metric learning is reliant on an effective objective function. As a result, various objective functions have been proposed, such as contrastive loss (Chopra et al., 2005) and triplet loss (Schroff et al., 2015). However, these loss functions have a specific limitation, i.e., they struggle to ensure that all samples from the same class will be pulled together in a common region within the representation space (Sohn, 2016). To address this limitation, Wen et al. (2019) proposed center loss, which adds a new regularization term to the softmax loss to pull samples to the corresponding class center.

Another important element of metric learning is the sampling strategy, i.e., the method of selecting samples for use in the training process. The sampling strategy can affect both the success and the training speed of deep metric learning. Schroff et al. (2015) used semi-hard mining, which selects triplets with negative samples that are nearly as close to an anchor as positive ones. Bucher et al. (2016) employed hard negative mining, which seeks hard triplets by choosing the most similar negative samples to an anchor. Meanwhile, Kumar et al. (2017) proposed smart mining, based on nearest neighbor samples to an anchor. In this paper, we follow Bucher et al. (2016) by using hard negative mining.

### 2.2 Imbalanced data

Imbalanced data occurs when the distribution of examples across classes in a dataset is biased or skewed. The distribution may vary from slightly biased to severe imbalance. Using imbalanced data in machine learning will cause the learning process to be biased toward the majority classes and to generalize poorly toward minority classes (Johnson and Khoshgoftaar, 2019).

Approaches to handle imbalanced data can be divided into two groups, i.e., data-level and algorithm-level. In terms of data-level approaches, a common method is to down-sample or decrease the number of samples in majority classes by randomly removing some of the samples (Pouyanfar et al., 2018). At the same time, the number of samples in the minority classes is increased or over-sampled, by duplicating samples or using data augmentation methods (Xie et al., 2019). These methods aim to even the contribution of each class to the learning process, and to eliminate the bias of the model toward particular classes. Regarding algorithm-level approaches, a common method is to weight losses, i.e., the loss weight of each sample is calculated according to the ratio of its class compared to other classes (Fernando and Tsokos, 2022). Another solution is to use models or objective functions that are insensitive to the imbalanced data (Huang et al., 2022).

### 2.3 Biomedical entity linking

Previous approaches to biomedical entity linking may be split into two groups, i.e., generative models and metric learning-based models. GENRE (Cao et al., 2020) is a representative example of a generative model, which firstly uses a sequence-to-sequence model to generate candidates, and then applies a classifier to re-rank the candidates. This method has a number of limitations. Firstly, it requires a large amount of annotated data for training. Secondly, when an entity has several synonyms, the use of a simple one-to-one mapping between mentions and entities is likely to result in sub-optimal performance. To address this issue, Yuan et al. (2022) proposed a two-step method using a transformer architecture. Specifically, as a pre-training step, they used a knowledge base (KB) to generate synonyms of entities in the input sequence to reduce the amount of training data needed. As a fine-tuning step, they used prompt tokens on the decoder side. Nevertheless, the training of all generative entity linking methods requires a significant amount of time and resources.

Most biomedical entity linking systems that take a metric learning approach carry out two steps, i.e., (1) learning representations of mentions and entities, and (2) ranking entity candidates using similarity scores. Fakhraei and Ambite (2018) proposed NSEEN, a Siamese network that maps mentions and entities to a feature space and uses the cosine metric to measure similarity between then. Agarwal et al. (2021) used two separate cross-encoders to learn the affinities between mentions and entities. They then employed a KNN-based clustering method to group mentions with high probabilities of referring to the same entity into clusters. Similarly to generative models, this method is costly in terms of computational cost and time, in both the training and inference phases.

## 3 Methods

**Figure 1:**
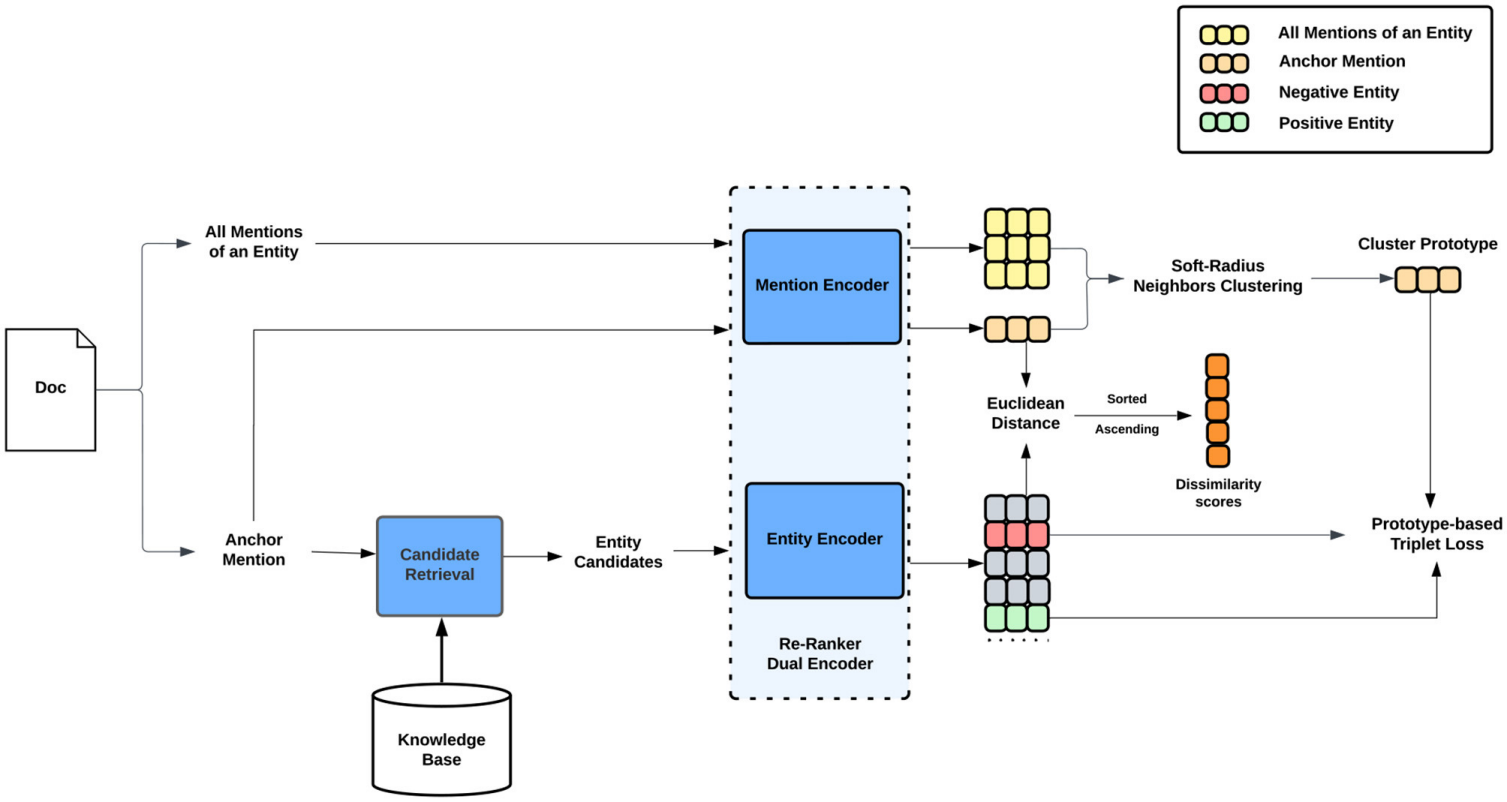
An overview of our framework. The system consists of two stages: Candidate Retrieval and Candidate Re-Ranking. Our proposed methods of prototype-based triplet loss and soft-radius neighbors clustering are applied at the candidate re-ranking stage. During this stage, the representations of anchor mentions and other mentions in their clusters as well as representations of candidate entities are updated concurrently by the prototype-based triplet loss. Euclidean Distance is used to measure the dissimilarity between a query mention and entity candidates. The final output entity is chosen by selecting the candidate that has the smallest dissimilarity score to the input mention.

Given a list of documents D∈𝒟, we denote $M^{d}=\left\{m_{1}^{d},m_{2}^{d},...,m_{n}^{d}\right\}$ as the set of *n* mentions in document *d* and a Knowledge Base (KB) of entities $ℰ=\left\{e_{1},e_{2},...e_{n}\right\}$. The purpose of entity linking task is to map each mention $m_{i}^{d}$ in document *d* to an entity *e*_*j*_ in KB ℰ. We break down the task into two stages, namely candidate retrieval and candidate re-ranking, as depicted in Figure 1. The first stage involves generating a list of entity candidates based on the cosine similarity between representations of mentions and entities. The second stage consists of re-ranking the list of possible entities using a dual-encoder architecture that scores the dissimilarity between mentions and entity candidates based on the Euclidean distance between their corresponding representations. We use prototype-based triplet loss and soft-radius neighbor clustering to concurrently update mention and entity representations. The final output is the candidate entity with the smallest dissimilarity score to the mention. The following sections provide detailed descriptions of the two stages.

### 3.1 Candidate retrieval

In a knowledge base KB, each entity is represented by a set of synonyms. Due to ambiguity, some synonyms may represent multiple entities. To handle these ambiguous synonyms, we create a dictionary of synonyms *S* = {*s*_1_:{*e*_1_, *e*_2_}, *s*_2_:{*e*_1_, *e*_3_}, ...}, where *s*_*i*_ is a synonym string and *e*_*j*_ is an entity represented by the synonym.

#### 3.1.1 Candidate representation

We represent mentions and synonyms in two ways, i.e., using both sparse and dense representation.

**Sparse representation:** We use tf-idf to obtain sparse representations of synonyms and mentions. Specifically, we use two tf-idf settings to generate two sets of candidates. The first setting involves calculating tf-idf based on character *n*-grams, in which *n*∈{2..5}. In the second setting, tf-idf is calculated at the word level with 1-grams. Words are tokenized by splitting on any non-alphanumeric characters, and stop words are removed before tokenizing. For both settings, all text is converted into lower case.

**Dense representation:** We use a deep transformer encoder (Vaswani et al., 2017) to obtain dense representations of synonyms and mentions. Specifically, we use the BERT architecture (Devlin et al., 2019) and initialize the weights from pre-trained SapBERT (Liu et al., 2020). In particular, we employ this model: cambridgeltl/SapBERT-from-PubMedBERT-fulltext from HuggingFace. Following the setting used by the authors, the output of the [CLS] token is used as the representation of the input. We only use the mention and synonym strings as input; this input is converted into lower case prior to feeding it into the model.

#### 3.1.2 Retrieval of candidates

All of the synonyms in the KB are scored according to their cosine similarity to a given mention, and then sorted in descending order. To obtain a list of candidates, we iterate over the sorted synonyms and append their entities to the list until we have *k* entity candidates.

### 3.2 Candidate re-ranking

In this section, we describe the dual-encoder architecture used to re-rank candidates, along with our proposed prototype-based triplet loss function and the soft-radius neighbor clustering.

#### 3.2.1 Model architecture

Our model architecture is inspired by Reimers and Gurevych (2019), but instead of using two symmetric BERT-based encoders, we use two separate ones: the *mention encoder* is used to encode mentions and their contexts, while the *entity encoder* is used to encode entities. We use pre-trained SapBERT (Liu et al., 2020) for both encoders and fine-tune the model using the prototype-based objective loss function.

**Mention encoder:** The mention encoder takes as input a sequence of tokens consisting of a mention and its context. The input sequence is formatted using BERT special tokens as follows:


$$\left[\text{CLS}\right]\;\left[c_{l}\right]\;\left[\text{START}\right]\;\left[m\right]\;\left[\text{END}\right]\left[c_{r}\right]\left[\text{SEP}\right]$$

where:

    *c*_*l*_ corresponds to tokens in the left context of the mention,
    *c*_*r*_ corresponds to tokens in the right context of the mention,
    *m* corresponds to tokens in the mention,
[START , [END]] are special tokens that indicate the position of mention in its context

The representation of a mention is obtained by taking average of the output embeddings of the [START] and [END] tokens.

**Entity encoder:** Similarly, we compose the input sequence for the entity encoder as follows:


$$\text{[CLS] [}type\text{] [SEP] [}syn_{\text{1}}\text{] [SEP] [}syn_{\text{2}}\text{] [SEP]}$$

where:

*type* is the entity semantic type extracted from the KB
*syn*_*k*_ corresponds to the tokens in a synonym of the entity

We use the entity type and the list of its synonyms as the entity description. To join synonyms, we employ the special token [SEP]. The representation of an entity is obtained from the output embedding of the [CLS] token.

#### 3.2.2 Prototype-based triplet loss

Triplet loss (Schroff et al., 2015) has been widely used as a loss function in metric learning. Given three sets of data points in the data set $x_{i}^{a},x_{i}^{p},x_{i}^{n}$ and their corresponding labels *y*_*a*_, *y*_*p*_, *y*_*n*_ such that *y*_*a*_ = *y*_*p*_ and *y*_*a*_≠*y*_*n*_. Usually, *x*^*a*^ are called anchor points, *x*^*p*^ and *x*^*n*^ are positive and negative points, respectively. The formula for triplet loss is:


(1) $${\left[∥f_{\theta }(x_{i}^{a})-f_{\theta }(x_{i}^{p}){∥}_{2}^{2}-∥f_{\theta }(x_{i}^{a})-f_{\theta }(x_{i}^{n}){∥}_{2}^{2}+\alpha \right]}_{+}$$

where *f*_θ_ is an embedding function and α is a margin that is enforced between positive and negative pairs. The embedding of each data point is represented as $f_{\theta }\left(x\right)\in {\mathbb{R}}^{d}$. The objective of this loss function is to learn the embedding function *f*_θ_ that results in the anchor $x_{i}^{a}$ being embedded to be closer to all other positive points $x_{i}^{p}$ than it is to any negative points $x_{i}^{n}$ in a *d*−dimensional Euclidean space.

When applying triplet loss to the entity linking task, we re-write the loss as follows:


(2) $${\left[∥f_{m}(m_{i})-f_{e}(e_{i}^{p}){∥}_{2}^{2}-∥f_{m}(m_{i})-f_{e}(e_{i}^{n}){∥}_{2}^{2}+\alpha \right]}_{+}$$

where *f*_*m*_ is the embedding function for mentions and *f*_*e*_ is the embedding function for entities. Unlike other classification tasks, in which positive points can be chosen randomly or intentionally from the same dataset with anchor points, entity linking tasks require that the entity associated with a mention is unique, meaning that the positive entity $e_{i}^{p}$ of an anchor *m*_*i*_ must be fixed during training. This makes the trained model very sensitive to imbalanced datasets. Randomly down-sampling to *k* samples for each class may remove useful information from the dataset. Moreover, in the standard triplet loss, the representation of each data point is learnt independently, without considering information about the distribution of mentions within a class. As a result, embeddings of mentions within a particular class tend to be located far away from each other in the feature space, which can cause the model to become stuck at poor local optima during training. To address these issues, we propose a new triplet loss function based on prototype.

Given a list of entities and its set of mentions in each document: *C* = {*e*_1_ = {*m*_1_, *m*_2_, *m*_3_}, *e*_2_ = {*m*_3_, *m*_4_}, *e*_3_ = {*m*_5_}, ...}, the prototype-based triplet loss is defined as:


(3) $$\begin{array}{l} {\left[∥\frac{1}{N}\sum f_{m}(m_{i}^{d})-f_{e}(e_{i}^{p}){∥}_{2}-∥\frac{1}{N}\sum f_{m}(m_{i}^{d})-f_{e}(e_{i}^{n}){∥}_{2}+\alpha \right]}_{+}\\ \;i\in [1..N],m_{i}\in M^{d},ℰ(m_{i})=e \end{array}$$

in which, $\frac{1}{N}\sum f_{m}\left(m_{i}^{d}\right)$ is the prototype of the cluster, *m*^*d*^ is a mention in document *d*.

In Equation (3), we assume that mentions referring to the same entity in a document are in the same cluster. As a result, the prototype is calculated by obtaining the average of all mention embeddings in the cluster. We use Euclidean distance as the distance metric to measure dissimilarity between mentions and entities. Using our proposed loss function, the distribution of all mentions in a cluster will be considered during training. Consequently, their embeddings are updated concurrently via the prototype such that they are close to their positive entity embedding and far away from the negative entity embedding. This makes the learning process more stable and effective, compared to the standard triplet loss. When using the standard triplet loss, the number of iterations in the training phase is equal to the number of mentions appearing in a document. However, by using the proposed loss, the number of iterations is reduced to the number of entities in a document, thus decreasing the number of training batches. Moreover, the proposed loss also makes use of all mentions in the data without down-sampling them, which addresses the issue of imbalanced data.

The means of selecting negative points for the triplet loss function has been shown to impact significantly on model performance in many studies (Xuan et al., 2020). To reduce the potentially huge number of possible negatives from the KB entities, we select negative points from a set of potential candidates. We use online hardest negative sampling technique (Chen et al., 2017) to choose the nearest negative candidate to mention *m*_*i*_ in the feature space, according to the current state of the model.

#### 3.2.3 Soft-radius neighbors clustering

**Figure 2:**
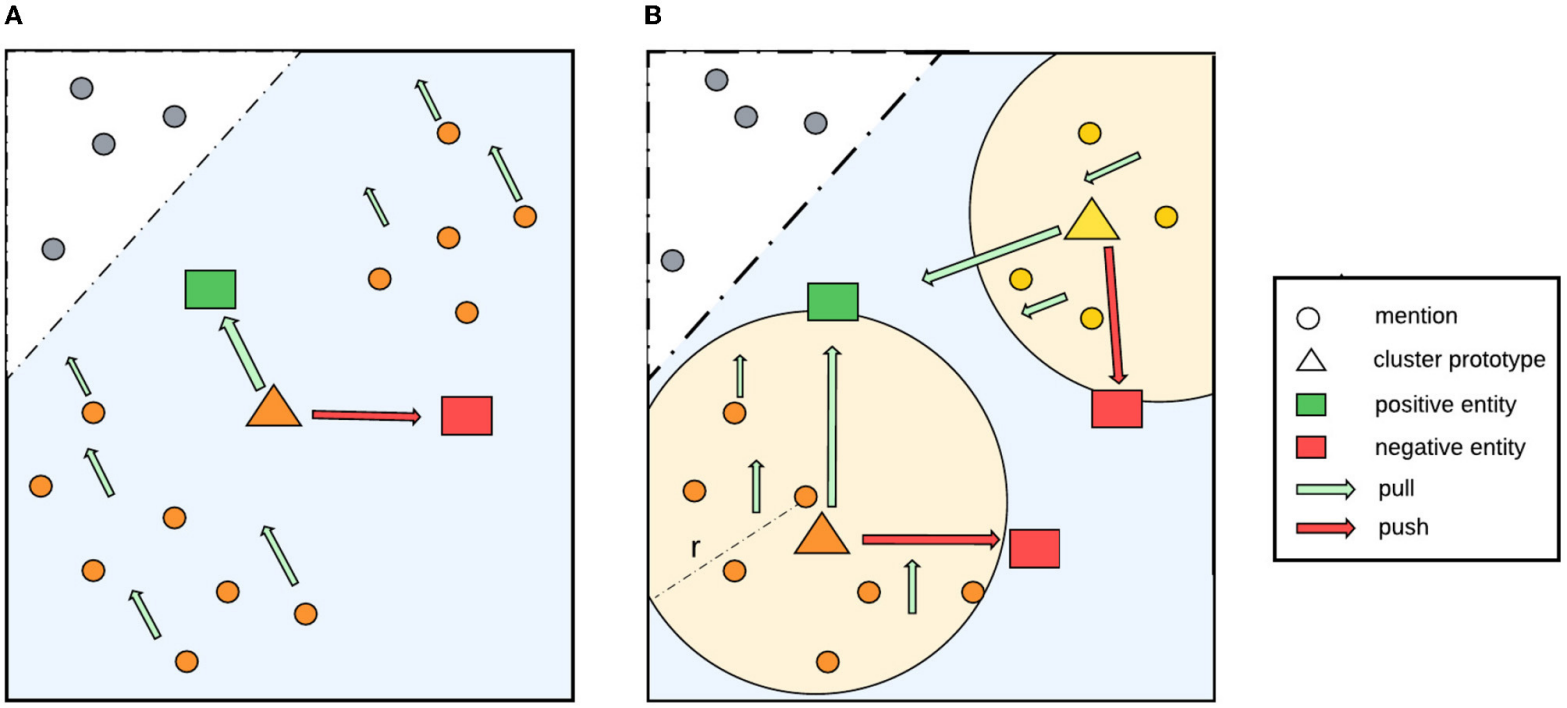
Our proposed method to detect latent clusters in a class. **(A)** Shows a case without clustering, in which the centroid of the whole class is already close to a positive entity and far away from a negative entity in the feature space. As a result, the loss might be zero, meaning that the model will learn nothing. **(B)** Shows that, by applying soft-radius neighbor clustering, the model obtains more accurate information about the distribution in the cluster and all the mentions in the clusters pull in the right direction toward the positive entity.

Within a document, different mentions that refer to the same entity may have diverse surface forms. This causes their corresponding embeddings to be located far apart from each other in the feature space, which is likely to reduce the effectiveness of our proposal. As illustrated in Figure 2A, without clustering, the prototype representation, i.e., the centroid of the whole class, is already close to a positive entity and far away from a negative one in the feature space. As a result, the loss might be zero, which causes the model to learn nothing. To address this limitation, we use a clustering algorithm based on radius neighbors (Bentley, 1975) to detect latent cluster in a class.

To detect the cluster of a given data point, we can simply use *k*-nearest neighbors, or kNN. However, when applying kNN in our scenario, there is a specific limitation. Since we have to fix the number of neighbors, i.e., *k*, we might include outlier data points (when a class has less than *k* instances) or exclude some data points (when a class has more than *k* instances). To alleviate the situation, we use radius neighbor clustering (Bentley, 1975) to locate all data points within a specific radius of a given data point.

Specifically, our soft-radius neighbor (SRN) clustering works as follows. Given a threshold radius λ and an anchor mention *m* in document *d*, we iterate over all other mentions that refer to the same entity as *m* in *d* and compute their distance to *m*. If the distance is smaller than the threshold λ, then the mention will be included in the cluster of *m*. Figure 2B illustrates that using this clustering, latent clusters can be detected within a class, which makes the representation learning process more accurate. As mentions will move in the same direction as the centroid during training, mentions in a cluster should be close to its centroid instead of being far away. It should be noted that distances between data points within a cluster tend to decrease during training because they keep moving increasingly close to its positive entity. To facilitate this behavior, we use a learnable parameter radius threshold λ, instead of a fixed one, which is the reason for the name *soft-radius*. The learned radius is the mean radius of all clusters in the training data. Additionally, we add the radius parameter to a fixed epsilon ϵ as a standard deviation to increase the coverage. The algorithm is shown in Algorithm 1.

**Algorithm 1:**
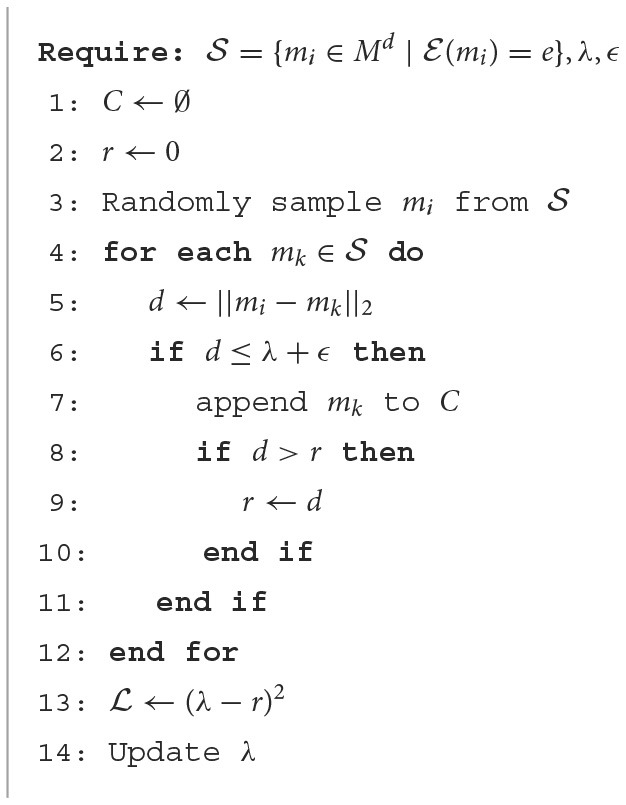
Form cluster and learning a radius λ.

## 4 Experiments and results

We conducted a series of experiments using the MedMentions (Mohan and Li, 2019) and BC5CDR (Li et al., 2016) datasets to evaluate the effectiveness of our proposed methods in addressing the imbalanced data issue in biomedical entity linking. We also compare our approach with our own baseline settings and other SOTA entity linking methods. We subsequently analyse the performance of our method by providing some examples to demonstrate the linking ability of our approach in comparison to the baselines. We also analyse prediction errors to show limitations of the proposed methods.

### 4.1 Datasets

**MedMentions** is a publicly available dataset that contains 4,392 titles and abstracts sourced from PubMed (Mohan and Li, 2019). Mentions in the dataset are annotated and linked to entities in the 2017AA full version of UMLS. As recommended by the creators of the dataset, we use the ST21PV subset, which uses a restricted set of 21 entity types. There are ~200,000 mentions in total, which are split into training (train), development (dev), and testing (test) sets. The dev and test sets contain a considerable number of entities that are unseen in the train set.

**BC5CDR** BioCreative V CDR (Li et al., 2016) is a challenge concerning chemical-induced disease (CID) relation extraction. The dataset contains 1,500 PubMed articles in which mentions are annotated and linked to MeSH.^1^ Mentions belong to one of two different entity types, i.e., *disease* and *chemical*, which makes it less challenging than MedMentions.

Table 1 reports statistics for each dataset, i.e., the total number of annotated mentions, the number of unique entities to which these mentions are mapped, and percentages of entities that are seen in the train set. The statistics reveal that MedMentions is a more challenging dataset than BC5CDR because a smaller percentage of the entities in the dev and test sets are seen during training.

**Table 1:** Statistics of annotations in the MedMentions and BC5CDR datasets.

	**MedMentions**			**BC5CDR**		
	**Train**	**Dev**	**Test**	**Train**	**Dev**	**Test**
Mentions	120 K	40 K	40 K	10 K	10 K	10 K
Entities	19 K	9 K	8 K	1.3 K	1.3 K	1.3 K
% seen	100	57.5	57.5	100	78.2	77.3

% seen indicates the percentage of entities seen in the train set.

**Figure 3:**
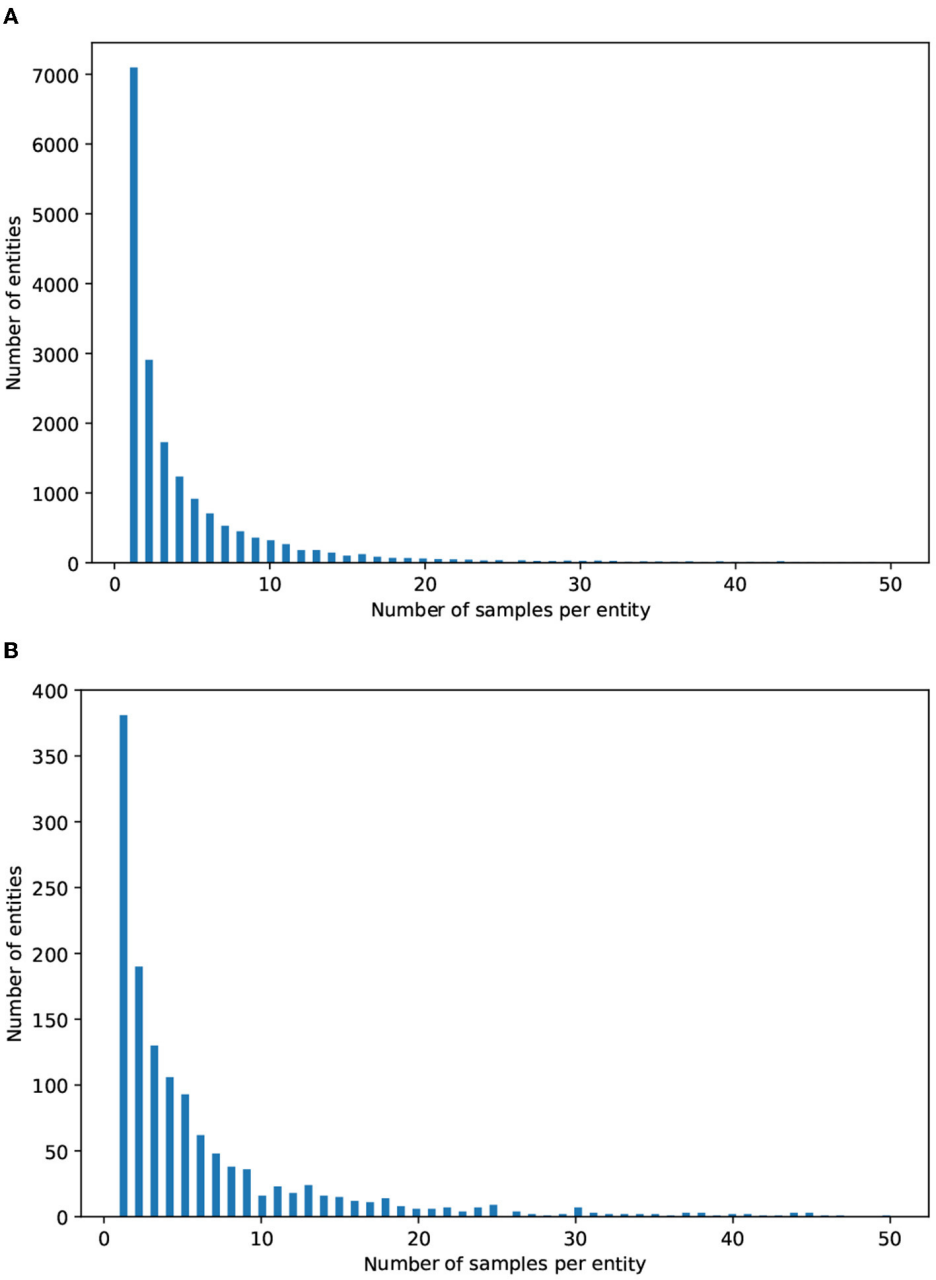
Distributions of the mentions per entity in **(A)** MedMentions and **(B)** BC5CDR. The x axis indicates the number of mentions per entity; for ease of readability, we only consider entities with 50 mentions or less. The y axis indicates the number of entities (i.e., concept classes).

As mentioned above, most real-world problems suffer from imbalanced data, especially in the biomedical domain. This is reinforced by Figure 3, which depicts the distribution of the number of mentions per entity in both datasets. It can be observed that a large number of entities have only 2 or 3 mentions, while a considerable proportion have between 4 and 10 mentions. There is a small number of entities that have more than 20 mentions.

### 4.2 Preprocessing

#### 4.2.1 Dataset

Each document in MedMentions and BC5CDR datasets is pre-processed as follows:

1. AB3P (Sohn et al., 2008) is applied to detect abbreviations in text, which are replaced with their corresponding full forms.
2. The text resulting from Step 1 is converted to lower case.
3. Documents are split into sentences using CoreNLP (Manning et al., 2014)
4. Sentences without any mentions are removed; the retained sentences containing mentions are converted into the IOB2 tag format. For MedMentions, we remove any overlapping mentions.

#### 4.2.2 Knowledge base

**UMLS:** We downloaded the full release of the 2017AA version of UMLS. We subsequently extracted all English synonyms of entities and their concept identifiers (CUIs) from the MRCONSO.RRF file. Each synonym is converted into lower case and added to a dictionary where the key is the entity CUI and the value is a list of distinct entity synonyms. As a result, the dictionary has ~3.4 M entities and 7.4 M synonym strings. For the entity type used in the entity encoder (Section 3.2.1), we extract the information from the MRSTY.RRF file.

**MeSH:** We use supplementary names and descriptor names as the synonyms of entities; other processing steps are the same as those described above for UMLS. The resulting dictionary contains 350 K entities with more than 950 K synonyms.

### 4.3 Settings

#### 4.3.1 Candidate retrieval

For the dense representation approach, we set the maximum input sequence length for BERT model to 50. Any longer sequence is truncated to 50 characters. For the sparse representation approach, there is no limitation on input length. To get the final *k* candidates, we merge two candidate lists obtained from two settings of tf-idf. We also remove any duplicate candidates from the final list.

In the retrieval phase, we use Faiss (Johnson et al., 2019) to store and index the vector representations of all the synonyms in the KB. We use an exhaustive search to find exact *k* nearest entity candidates.

#### 4.3.2 Candidate re-ranking

To evaluate the effect of the prototype-based loss and the soft-radius neighbor (SRN) clustering, we use standard triplet loss as one of our baselines. Regarding the ability to handle imbalanced data, we use data-level approaches as our baseline. In particular, we choose down-sampling instead of over-sampling as over-sampling will significantly increase the amount of data, hence increasing the computational cost in the training phase. This goes against the purpose of our work which reduces the computational cost while retaining competitive performance. The detail of our baselines is as follows:

1. **Triplet loss with all samples**: We trained the dual encoder using the standard triplet loss on all data samples.
2. **Triplet loss with down-sampling**: This is similar to the first setting, except that we apply down-sampling to reduce the imbalance in the dataset. The down-sampling is applied at the document level, by randomly choosing one sample per class in each document.
3. **Prototype-based triplet loss**: This is also similar to the first setting, apart from the use of prototype-based triplet loss.
4. **Prototype-based triplet loss with SRN**: The same as setting 3, but with the addition of SRN clustering.

To assess whether the performance of each setting in the aforementioned steps exhibits statistical superiority over the others, we employ the McNemar's test (Dror et al., 2018) with a significance level set at 0.05.

### 4.4 Implementation details

We implemented our systems using the Pytorch (Paszke et al., 2019) framework. All experiments were run on a NVIDIA A100 GPU. For the candidate re-ranking step, the maximum sequence length of BERT is set to 128, and any longer input sequence is truncated to 128. The model was trained using Adam optimizer (Kingma and Ba, 2015), with a learning rate of 10^−5^ and a batch size of 32. The margin α in our loss function is set to 1.2 for MedMentions and 1.6 for BC5CDR. The threshold radius parameter λ is initialized to 11.0 and the epsilon ϵ is set to 1.0. The learning rate to update λ is set to 0.005 for MedMentions and 0.01 for BC5CDR. We train three epochs for MedMentions and six epochs for BC5CDR. Apart from the number of epochs, we use the same hyperparameters for both datasets.

We use top 1 accuracy (Acc@1) as our evaluation metric, following previous work on biomedical entity linking tasks (Agarwal et al., 2021; Bhowmik et al., 2021; Wawrzik et al., 2023). We define Acc@1 as being correct if the ground truth entity is predicted by our model in the top 1 prediction, otherwise it is incorrect.

### 4.5 Results

#### 4.5.1 Candidate retrieval

The results of this step are important, since they will affect the candidate re-ranking step. Specifically, a high recall in this step will allow the re-ranking model to achieve better performance. Table 2 reports the average recall considering different numbers of candidates (*k*), i.e., whether or not the gold entity is included in top *k* candidates for a given mention. As illustrated in the table, dense representations generally resulted in higher recall than sparse representations, with the exception of recall@2 and recall@4 in MedMentions. In common with previous work, we use 64 candidates as input to the re-ranking model. In this case, the recall for MedMentions was above 87% and above 95% for BC5CDR. Results from the statistical significance test suggest that when *k* is set to 64, the recall is significantly better than those by the others *k* in both MedMentions and BC5CDR datasets. We however note that recall@64 by dense representation is significantly better than that by sparse representation on BC5CDR but not on MedMentions.

**Table 2:** Candidate retrieval recall on MedMentions and BC5CDR dataset.

**Recall@*k***	**MedMentions**		**BC5CDR**	
	**SR**	**DR**	**SR**	**DR**
1	46.3	47.0	87.0	88.7
2	66.5	61.4	90.1	92.6
4	73.3	73.0	91.3	95.0
8	77.7	80.2	92.5	96.3
16	80.7	83.1	93.7	97.5
32	83.4	85.4	94.1	98.0
64	87.1	**87.2** ^*^	95.2	**98.5** ^*^

“SR” stands for sparse representation and “DR” stands for dense representation. ^*^Indicates statistical significance compared to the others (with *p*-value < 0.05). The bold values indicate the result of the best performing system.

#### 4.5.2 Candidate re-ranking

Table 3 compares entity linking performance on the development sets of MedMentions and BC5CDR using the two candidate retrieval approaches, and reveals different trends. On BC5CDR, the re-ranking model achieved the best accuracy when using dense representation across all four settings. In contrast, on MedMentions, use of the sparse representations resulted in the best accuracy across all four settings. We hypothesize that this situation was caused by the performance gap of recall@64 between the sparse and dense representations, as shown in Table 2. In the case of BC5CDR, recall@64 with the dense representations was 3 points higher than when sparse representations were used. However, in the case of MedMentions, the gap between the two representations is only 0.8 points. As a result, and in contrast to BC5CDR, the overall performance on MedMentions was not improved by using dense representation.

**Table 3:** Acc@1 produced by four settings on the development set of MedMentions and BC5CDR.

**Models**	**Candidate retrieval**	**MedMentions**	**BC5CDR**
Triplet loss + All samples	SR	70.7	85.8
	DR	70.3	87.8
Triplet loss + Down-sampling	SR	71.6	86.1
	DR	71.3	88.1
Prototype-based Triplet loss	SR	71.5	86.2
	DR	71.4	88.2
Prototype-based Triplet loss + SRN	SR	**71.9** ^*^	87.0
	DR	71.7	**88.9** ^*^

Emboldened numbers indicate the best performing system, while underlined ones indicate the second best. “SR” stands for sparse representation. “DR” stands for dense representation. ^*^Indicates statistical significance compared to the others (with *p*-value < 0.05).

In general, the prototype-based triplet loss with SRN setting outperforms the second best baseline setting by 0.3 accuracy points on MedMentions and 0.7 points on BC5CDR. We can see that imbalanced dataset affects the performance of model in the basic setting (i.e., Triplet Loss + All samples). The use of down-sampling boosts the accuracy on both datasets. Using the prototype-based triplet loss produced mixed results: the accuracy was improved for BC5CDR, but not for MedMentions. However, combining this loss with SRN clustering resulted in the best performance for both datasets. Results from the statistical tests indicate that such performance was significantly better than the others.

Without the use of down-sampling, the model using the standard triplet loss suffered from over-fitting in the first two epochs on MedMentions. However, as mentioned previously, down-sampling can cause information loss, which generally impacts upon the model performance, especially in representation learning. Furthermore, for biomedical entity linking, it is crucial that the model can learn representations of mentions that occur in different contexts as well as those having different surface forms. The combination of prototype-based triplet loss with SRN clustering preserves this information for the use by the model. Consequently, it is able to learn representations of more synonyms of an entity while keeping the contribution of each class balanced in the training process. This explains why this setting was able to outperform the Acc@1 of the baseline setting, as shown in Table 3.

We applied the setting that performed best on development set (i.e., prototype-based triplet loss with SRN clustering) to the test set and compare the results to those obtained by SOTA methods in Table 4. In this table, results are cited from the corresponding papers. To make the comparison fair, we note two points: (1) in the case of Angell et al. (2021), we reported the results without entity gold types, and (2) in the case of SapBERT (Liu et al., 2020), we collected the numbers from Zhang et al. (2022). It can be seen that our model outperforms most previously proposed methods on both BC5CDR and MedMentions. Compared to KRISSBERT (Zhang et al., 2022), our approach resulted in better accuracy on MedMentions but slightly lower accuracy on BC5CDR. In contrast, compared to the clustering-based method developed by Angell et al. (2021), our method performs better on BC5CDR but exhibits lower performance on MedMentions. Overall, our method achieves the second best performance system across both datasets, indicating greater stability than other SOTA methods.

**Table 4:** Performance (Acc@1) of our method in comparison with SOTA models on the test sets of MedMentions and BC5CDR.

**Models**	**MedMentions**	**BC5CDR**
SapBERT (Liu et al., 2020)^*^	44.2	89.9^+^
Angell et al. (2021)	**74.1**	91.4
Agarwal et al. (2021)	72.3	–
KRISSBERT (Zhang et al., 2022)	70.6	**93.8** ^+^
Prototype-based Triplet Loss + SRN (Ours)	72.8	93.5

Emboldened results indicate the best performing system, while underlined results denote the second best system. ^*^The results were sourced from Zhang et al. (2022). ^+^We averaged the performance for disease and chemical entities. The bold values indicate the result of the best performing system.

Similarly to Angell et al. (2021), we also report the performance of our method on seen and unseen entities in Table 5. As expected, our proposed model did not perform as well as Angell et al. (2021)'s system on both seen and unseen entities of MedMentions, especially on unseen ones. However, our system outperformed theirs on entities of BC5CDR with a large margin on unseen entities.

**Table 5:** Linking accuracy on seen and unseen entities.

**Model**	**MedMentions**		**BC5CDR**	
	**Seen**	**Unseen**	**Seen**	**Unseen**
Angell et al. (2021)	**77.3**	**62.9**	94.9	73.8
Ours	76.5	58.1	**96.1**	**80.1**

The bold values indicate the result of the best performing system.

We additionally report the training and inference time of our method on MedMentions compared to SOTA methods in Table 6. It can be observed that our method exhibits significantly reduced training and inference times in comparison to other methods, while maintaining a competitive accuracy. The training time is reduced according to the lower number of training batches. As explained in Section 3.2.3, all mentions within a cluster are fed to the model concurrently rather than individually. The lower inference time of our model is due to our use of a dual encoder, which separately encodes representation of mentions and entities. This means that entity candidates are only encoded once and are saved for later retrieval.

**Table 6:** Training time, inference time, and accuracy of our method compared to two SOTA method.

**Training**	**Time (hours)**	**Inference**	**Time (hours)**	**Acc@1**
Angell et al. (2021)	72	kNN Graph	4	74.1
Agarwal et al. (2021)	32.1	Independent	1.5	72.3
Prototype-based Triplet loss + SRN	10	Independent	0.5	72.8

## 5 Discussion

### 5.1 Impact of imbalanced data

To better understand the impact of imbalanced data on our approach to entity linking, we analyse some seen mentions from minority classes in the test set. Note that *seen* means only that the ground truth entity to which the mention is linked also appears in the training data; the actual surface form of the mention in the test set may not occur in the training set. The types of example mentions shown in Table 7 are fairly representative of biomedical text, in which different mentions of entities can be diverse in terms of their surface forms, as well as being ambiguous. The results show that, despite the fact that the mentions shown are linked to entities seen in the training data, the re-ranker model using triplet loss without down-sampling (TLA) exhibits lower performance than other baseline models. This can be explained by the model's bias toward the majority class entities, whose synonyms appear many times in the training data. Although the application of down-sampling (TLD) results in better performance than TLA, the removal of samples by this technique means that the model is unable to adequately learn the representation of mentions in different contexts or different surface forms of mentions. This explains why the use of down-sampling (TLD) resulted in some incorrect predictions (see Examples 2 and 3). In contrast, the use of prototype-based loss and SRN (PTL+SRN) helped the model to make correct predictions in all cases.

**Table 7:** Examples of entity linking results output by the four different settings of the re-ranker.

**No.**	**Mentions**	**Candidates**		**Re-ranker**	**Predict**
		**Entity ID**	**Synonyms**		
1	... experience with **abnormal ocular motility** in patients treated with subtenon carboplatin chemotherapy.	**D015835**	**Internuclear ophthalmoplegias eye movement disorder**	TLA	D004409
		D004409	Medication-induced dyskinesia	TLD	**D015835**
		D002925	Ciliary motility disorders dyskinesia	PTL	**D015835**
		...	...	PTL + SRN	**D015835**
2	... the patient's **sinus bradycardia** and the drug interaction between metoprolol and terbinafine.	D004409	Medication-induced dyskinesia	TLA	D054138
		D054138	Sinus arrest, cardiac	TLD	D054138
		**D012804**	**Sick sinus node syndrome**	PTL	**D012804**
		...	...	PTL + SRN	**D012804**
3	... early **postoperative delirium** incidence risk factors were then assessed through three different multiple regression models.	D020250	Postoperative nausea and vomiting	TLA	D003693
		D003693	Delirium, subacute	TLD	D020250
		**D011183**	**Emeses, postoperative; complications, postoperative**	PTL	**D011183**
		...	...	PTL + SRN	**D011183**
4	... because of aplastic crisis with septicemia and marked abnormalities in liver function and died of **hemorrhagic bronchopneumonia**.	D006468	Hemopneumothorax	TLA	**D001996**
		**D001996**	**Bronchial pneumonia**	TLD	**D001996**
		D011002	Pleuropneumonias, contagious; tuberculosis sanitorias	PTL	D006468
		...	...	PTL + SRN	**D001996**

TLA, Triplet Loss + All samples; TLD, Triplet Loss + Down-sampling; PTL, Prototype-based Triplet Loss; PTL+SRN, Prototype-based Triplet Loss + SRN. The emboldened IDs and synonyms in the lists of candidates correspond to the ground truth entities.

### 5.2 Visualizing representations

**Figure 4:**
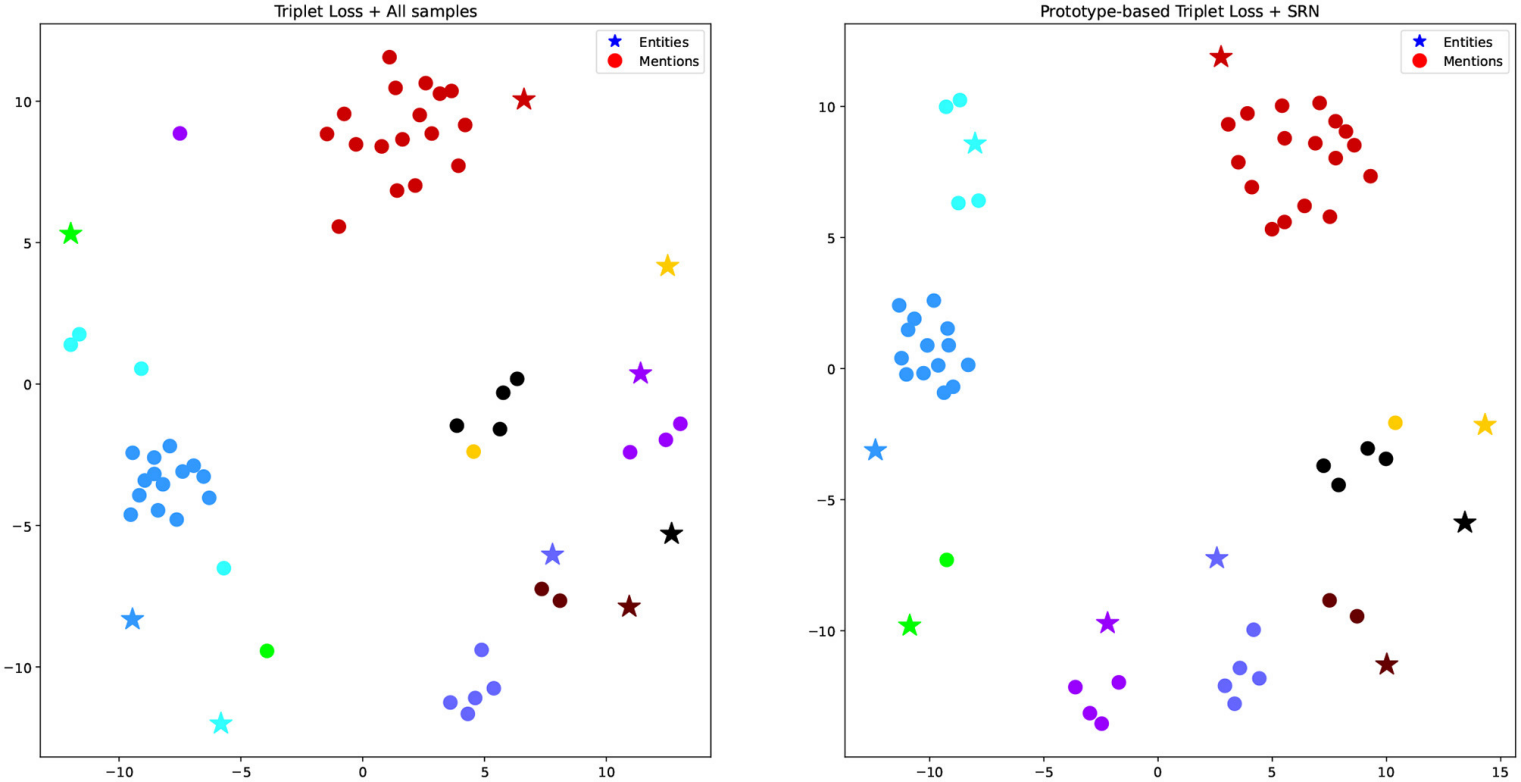
Multi-dimensional scaling representation of examples and their corresponding entities in two experiments. Mentions referring to the same entity have the same color.

To test our hypothesis that mention representations learned from traditional triplet loss may not appear close to their ground truth entities, especially in imbalanced datasets, Figure 4 visualizes some mention representations and their corresponding ground truth entities produced by the dual encoder in two settings, i.e., Triplet Loss + All samples (left) and Prototype-based Triplet Loss + SRN (right). We used Multi-dimentional Scaling (Torgerson, 1952) to map high dimensional representations into 2D ones. In the figure, stars correspond to entity representations, while dots correspond to mention representations. Mentions referring to the same entity have the same color. In most cases, entity representations are located far away from their corresponding mention representations. However, mention representations on the right figure (Prototype-based Triplet Loss + SRN) are clustered better than those on the left one (Triplet Loss + All samples). Specifically, in the left figure, mentions of the yellow, green, black, and cyan entities are far away from each other and often occur close to other entities. Meanwhile, in the right figure, all mentions occur in a relatively close proximity to their ground truth entities and there is a better degree of separation from other entities. This illustrates that the Prototype-based Triplet Loss + SRN model is able to learn better representations compared to the baseline, which helps to explain its superior performance shown in Table 3.

### 5.3 Limitations

There are two potential limitations of our proposed Prototype-based Triplet Loss + SRN model. Firstly, while the representations of mentions in a cluster are updated concurrently to be closer to their ground truth entity, the model also pushes mentions far away from hard negative entities of an anchor. This may affect mentions of other entities by inadvertently pushing them closer to their hard negative ones. Therefore, it is important to select suitable negative samples as well as anchor mentions. We believe that this issue could be alleviated by intentionally choosing an anchor close to the center of a cluster instead of choosing it randomly. We will investigate this further as future work.

Secondly, since we only focus on the second stage of entity linking, i.e., candidate ranking, the retrieval of candidates was performed using only fairly simple approaches based on statistic and pre-trained neural networks. Although high recall of candidates was achieved for BC5CDR, there remains considerable room for improvement for MedMentions. Accordingly, follow-up work could explore the use of dual encoder architecture to generate candidates (Gillick et al., 2019; Agarwal and Bikel, 2020).

## 6 Conclusion

In this article, we have introduced a metric learning-based approach using BERT-based dual encoders to address the problem of imbalanced data in biomedical entity linking. Specifically, we propose a loss function named prototype-based triplet loss, combined with soft-radius neighbor clustering, to learn the representations of mentions and entities. Our proposed method can partially resolve the issue of imbalanced data, because it considers each class in a document as a whole, regardless of the number of instances in the class. Experimental results on two biomedical datasets demonstrate that our method is able to significantly reduce computational cost in the training and inference phases, compared to other SOTA methods, while also maintaining competitive accuracy with these methods. We believe that our method may also be useful for application in other domains that have imbalanced datasets.

## Data availability statement

Publicly available datasets were analyzed in this study. This data can be found at: https://github.com/chanzuckerberg/MedMentions/blob/master/st21pv/ReadMe.md; https://biocreative.bioinformatics.udel.edu/tasks/biocreative-v/track-3-cdr/.

## Author contributions

NL conceived and designed the framework and carried out the implementation and experiments. NN was involved in planning and supervising this work. All authors discussed the results and contributed to the final manuscript.

## Footnotes

^1^ https://www.ncbi.nlm.nih.gov/mesh
